# Supplementary material for: Confirmation of ‘Pollen- and Seed-Specific Gene Deletor’ System Efficiency for Transgene Excision from Transgenic Nicotiana tabacum under Field Conditions
Source: Int J Mol Sci. 2023 Jan 6;24(2):1160. doi: 10.3390/ijms24021160 (PMC9866632; doi:10.3390/ijms24021160)
Supplement: Supplementary file 1 [file ijms-24-01160-s001.zip › ijms-2091536-supplementary.pdf]

Supplementary Figures:

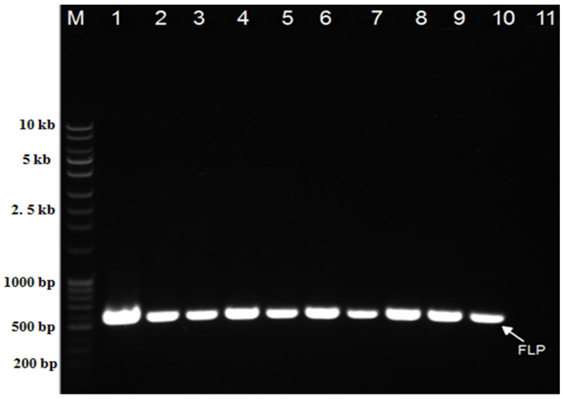

Figure S1: PCR amplification of FLP gene. M: Marker; 1: Plasmid DNA (Positive Control); 2-10: Transgenic tobacco lines; 11: Wild Type (Negative Control);

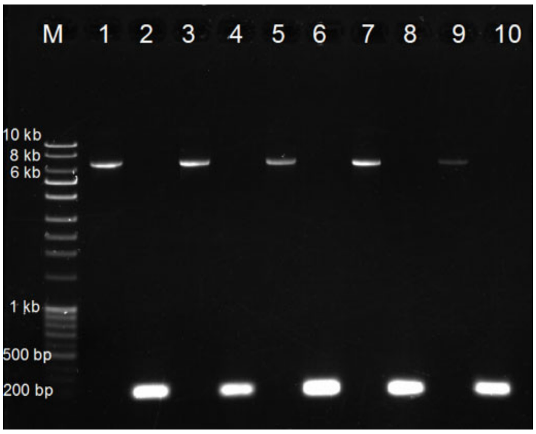

Figure S2. Electrophoresis gel showing the PCR products amplified using LF-specific primers from the T0 (lane no: 1, 3, 5, 7 and 9) and T1 (lane no: 2, 4, 6, 8 and 10) generations of field-grown transgenic plants carrying the 'pollen-and-seed-specific Gene-Deletor' cassette. The 0.2-kb DNA sequence fragment was PCR-amplified using genomic DNA from the self-pollinated, field-grown T1 seedlings produced by line D31. Lane M: Marker; Lanes 1–2: line D4; lanes 3–4: line D10; lanes 5–6: line D31; lanes 7–8: line D56; lanes 9–10: line D43.

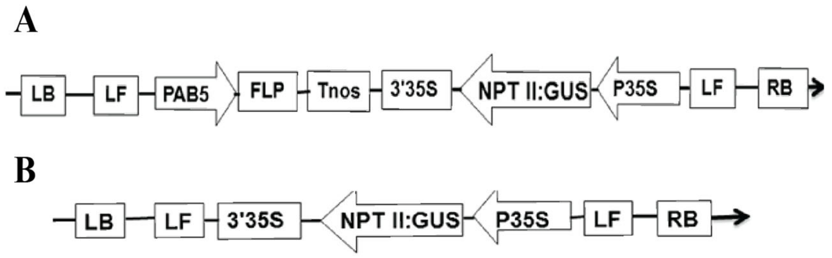

Figure S3. Schematic showing the (A) 'pollen-and-seed-specific Gene-Deletor' cassette. LB and RB: left- and right-borders of the T-DNA, respectively; LF: fused loxP and FRT (loxP-FRT) recognition sequence; PAB5: pollen-and-seed-specific *Arabidopsis* PAB5 gene promoter; P35S: CaMV 35S gene promoter; Tnos: terminator of the *Agrobacterium tumefaciens* nopaline synthase gene; FLP: FLP recombinase gene; GUS: β-glucuronidase gene; NPTII: neomycin phosphotransferase II gene (kanamycin resistance as plant selection marker); (B) control cassette. All transgenes were flanked by fused loxP-FRT sequences.

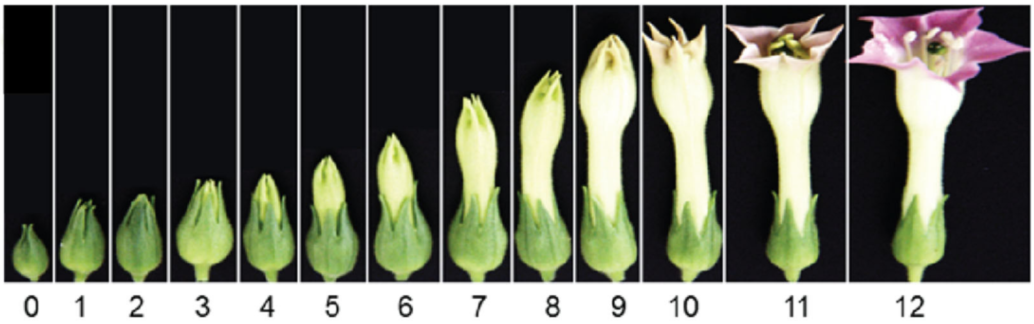

Figure S4. *Nicotiana tabacum* flower developmental stages (as proposed by Goldberg, 1988). We modify scale of maturity stages to include stage 0 before stage 1.
